# Differences between Chinese Adolescent Immigrants and Adolescent Non-Immigrants in Hong Kong: Perceived Psychosocial Attributes, School Environment and Characteristics of Hong Kong Adolescents

**DOI:** 10.3390/ijerph18073739

**Published:** 2021-04-02

**Authors:** Hechao Jiang, Daniel T. L. Shek, Moon Y. M. Law

**Affiliations:** 1 Research Institute of Social Development, Southwestern University of Finance and Economics, Chengdu 611130, China; jhchao@swufe.edu.cn; 2 Department of Applied Social Sciences, The Hong Kong Polytechnic University, Hong Kong SAR 999077, China; 3 Department of Applied Social Sciences, HKCT Institute of Higher Education, Hong Kong SAR 999077, China; moonlaw@ctihe.edu.hk

**Keywords:** adolescent immigrants, adolescent non-immigrants, Hong Kong adolescents, psychosocial attributes, perceived school environment, social perception

## Abstract

Although the impact of immigration on adolescent developmental outcomes has received extensive scholarly attention, the impact of internal migration, particularly in the Chinese context, on adolescents’ psychosocial development has not been scientifically investigated. This study examined whether mainland Chinese adolescent immigrants (N = 590) and adolescent non-immigrants (*n* = 1798) differed on: (a) psychosocial attributes indexed by character traits, well-being, social behavior, and views on child development, (b) perceived school environment, and (c) perceptions of characteristics of Hong Kong adolescents. Consistent with the healthy migration hypothesis, Hong Kong adolescents and mainland Chinese adolescent immigrants did not differ on most of the outcomes; Chinese adolescent immigrants showed higher perceived moral character, empathy, and social trust than did Hong Kong adolescent non-immigrants. Chinese adolescent immigrants also showed more favorable perceptions of the school environment and moral character, social trust and social responsibility of adolescents in Hong Kong. This pioneer Chinese study provides support for the healthy immigration hypothesis (immigration paradox hypothesis) but not the immigration morbidity hypothesis within the specific sociocultural context of Hong Kong in China.

## 1. Introduction

The number of mainland Chinese migrant adolescents in Hong Kong has rapidly grown in the past decades. By the end of 2018, immigrants aged 0–14 from mainland China accounted for 12.36% of Hong Kong’s population in 2018 [1]. Extensive research has shown that the process of migrating was stressful [2,3,4,5,6]. As adolescence is full of challenges [7,8,9,10], adolescents are more likely to be victims of immigration [11]. However, evidence on whether there are psychosocial adjustment differences between immigrant adolescents and non-immigrant adolescents is conflicting [12,13].

Theoretically, there are two different perspectives about the psychosocial consequences of immigration. On the one hand, the migration morbidity hypothesis states that immigration leads to psychosocial problems in immigrant populations [14], which is supported by empirical evidence. Comparing with native adolescents, higher internalizing problems were reported by immigrants in Norway [15,16], Switzerland [17], The Netherlands [18], Italy [4], Denmark [19], Austria [20] and Europe [21]. In addition, higher externalizing problems in immigrants in Dutch [22], Spain [23], and Israel [24] were reported. On the other hand, the migration paradox hypothesis or healthy immigration hypothesis posits that migration does not necessarily lead to poorer psychological or behavioral outcomes because migration experience may induce growth in immigrant adolescents and they are protected by protective factors such as more educational opportunities, good social networks and social support [25]. Studies suggested that adolescent immigrants in America [11,26], Austria [14] and The Netherlands [27] showed fewer internalizing symptoms and externalizing problems than did adolescent non-immigrants; immigrants in Sweden [28] and Austria [3] also showed no difference in mental health problems compared to native adolescents.

There are several limitations of the research surrounding the effect of migration on adolescent development. First, existing studies are mostly Western studies with non-Western immigrants migrating to Western countries (i.e., international migration). Comparatively speaking, studies on immigration with specific reference to Chinese people are scarce. In view of the large size of the Chinese population (roughly 1.4 billion people), there is a need to understand the impact of migration within different Chinese communities. In fact, this is an argument commonly raised in Chinese adolescent research [29,30].

Second, comparing with international migration, there is less attention paid to migration within a country (i.e., internal migration). As internal migration does not change the immigrants’ living environment as significantly as international migration does, internal migration may not result in poorer psychological and behavioral adaptation outcomes. For example, with specific reference to Hong Kong (a Special Administrative Region in China), a Tam and Lam’ study [31] revealed that compared to their native counterparts (*n* = 750), Chinese migrants (*n* = 243) not only did not have higher perceived stress, but they were able to cope with stress more positively, and presented less delinquent behavior and higher self-esteem. Leung and Karnilowicz [32] compared adaptation of Chinese adolescents in Hong Kong and Australia, and argued that immigrant adolescents who resided in Australia reported lower sociocultural adaptation than did those in Hong Kong.

Third, existing studies have primarily focused on mental health outcomes of migration. Some studies explored specific mental health problems in immigrants, such as depression [15,33,34], anxiety [3,14,35], self-esteem problems [4,24,27,36] and externalizing problems (e.g., alcohol abuse, delinquency and aggressive behavior [16,17,22,24,37,38]). In fact, except for some isolated studies [16,22,28,39,40], few studies have evaluated multiple adolescent behavioral problems of immigrant and non-immigrant adolescents.

From a holistic conception of adolescent development, it is argued that researchers should compare adolescent immigrants and non-immigrants on other developmental domains besides negative mental health. First, as character traits (such as materialism, egocentrism and moral character) are important aspects of adolescent development [10], we should understand whether immigrants and non-immigrants differ on the related measures. For example, because economic disadvantage commonly occurs in immigrant adolescents, they may be more materialistic. Second, as adolescent well-being such as social well-being (e.g., empathy) and individual well-being (e.g., life satisfaction) are key adolescent qualities of life indicators [8,9,41], it is necessary to examine the related differences in well-being indicators, particularly with reference to the positive youth development framework. Third, adolescent social behavior, including social trust, sense of responsibility, prosocial behavior and social behavior, are important adolescent developmental outcomes [10]. For example, according to the migration morbidity hypothesis, adolescent immigrants may have a lower sense of social trust than do adolescent non-immigrants. Unfortunately, previous studies have focused more on the effects of migration on individual problem behavior, such as alcohol abuse, delinquency and aggressive behavior [17,18,22,24,37,38] while very few studies focused on the social behavior of immigrants. Fourth, it is theoretically interesting to understand whether immigrants and non-immigrants differ in their views on child development, such as whether children have to win as early as possible and whether excellent academic result is more important than moral character (i.e., instrumental beliefs). As immigrants have to climb up the social ladder, it is a common belief that they would place strong emphasis on academic excellence as a means to climb up the social ladder.

In addition to the above psychosocial attributes, as school is an important developmental context for adolescents, how adolescent immigrants view their school environment (such as school climate, teaching techniques of teachers, moral education in school, and life skills education in school [30]) would have theoretical and practical implications. A common myth is that immigrant adolescents would experience school difficulties and they view school environment in a negative light [13,42,43].

Finally, few studies have compared the social perceptions of immigrant and non-immigrant students such as perceptions of adolescents in general. Social perception is essential for the integration of migrants into local communities. Assimilation theory suggests that immigrant adolescents usually try to align their attitudes and behaviors with the locals when they arrive in the receiving community [44,45], but this is assuming that immigrant adolescents having clearer perceptions of the local adolescents’ psychosocial situation. Limited research suggests that psychosocial adjustment of immigrant adolescents is the process of adapting attitudes and behaviors of non-immigrant adolescents [2,37,46], but those studies lack a comparison of social perception differences between immigrants and non-immigrant adolescents.

Regarding the conflicting findings on the impact of migration on adolescent developmental outcomes, we should examine several confounding factors. First, there is the negative effect of poverty on adolescent developmental outcomes. Findings based on the European Social Survey suggested that low socioeconomic status, not immigration, explains the higher risk among immigrants [46]. Second, gender moderates the impact of migration on adolescent developmental outcomes. For example, Dimitrova and Chasiotis [47] found that immigrant boys obtained higher scores than did girls on emotional instability and aggression, but lower scores than girls on prosocial behavior. Third, psychosocial outcomes of migration may vary across generation status. Sam et al. [48] found that first-generation and second-generation differed in psychological and sociocultural adjustment among immigrant adolescents in Norway. Moreover, Alati et al. [3] found no significant differences in mental health between second-generation immigrant adolescents and natives in Australia.

Against the above background, we attempted to examine differences between mainland Chinese migrant adolescents and native adolescents in Hong Kong in terms of a wide range of developmental outcomes, including perceived psychosocial attributes (character traits, well-being, social behavior and views on child development), perceived school environment, and perceptions of Hong Kong adolescents. These six domains are crucial aspects of youth positive development revealed by many studies in developmental psychology [8,9,10,30,41]. In fact, perceived psychosocial attributes, such as well-being and social perceptions, are also important outcomes providing reference points for behavior (i.e., social norms) and social integration. Hence, comparing immigrants and non-immigrants in these six domains will help to remedy the shortcomings of previous studies that focused on only one domain (e.g., negative mental health). This strategy also helps to evaluate the immigration morbidity hypothesis and immigration paradox hypothesis in a comprehensive manner. To remove the confounding effects of background sociodemographic factors, we controlled age, gender, paternal education level, maternal education level, family intactness, and family economic status in the analyses. Our general research question is: Do adolescent immigrants from mainland China differ from adolescent non-immigrants in Hong Kong in terms of psychosocial attributes, perceived school environment and perceived characteristics of Hong Kong adolescents? According to the migration morbidity hypothesis, migrant adolescents would perform worse than did non-migrant adolescents on the related measures. On the other hand, according to the healthy migrant hypothesis, migrants would have more positive perceived outcomes than did non-migrants and/or there would not be differences between the two groups.

## 2. Materials and Methods

### 2.1. Participants and Procedure

The Wofoo Foundation supported this collaborative study. Through stratified cluster sampling method, 20 local secondary schools were recruited. In the selected schools, we randomly selected three to five classes to join the study. Students were invited to complete a paper-and-pencil questionnaire measuring their moral character, psychosocial development, prosocial behavior, parenting and school-related factors [10,49]. The project was approved by the “Human Subjects Ethics Sub-Committee” in the affiliated university of the corresponding author. We had obtained written consent from the participating schools, student participants, and their parents prior to data collection.

Originally, 2747 students completed the questionnaire. Among these participants, 1798 were non-immigrant students, 590 were Chinese immigrant students, 39 were immigrant students from other countries, and 320 students did not indicate their status as immigrant or non-immigrant. As the aim of this study was to investigate the differences between mainland Chinese immigrant students and local Hong Kong non-immigrant students, the 320 students who did not indicate their status were removed from the analyses, the 39 immigrant students (i.e., international immigration) were also removed from the analyses, and the final participant number of the study was 2388. The mean ages were 14.23 ± 1.68 years old for the non-immigrant group and 15.14 ± 1.87 years old for the immigrant group. There were 51.2% female students in the non-immigrant group and 57.7% female students in the immigrant group. Table 1 presents the demographic characteristics of the two groups.

### 2.2. Measures

The questionnaire consisted of different sections measuring character traits, perceived school environment and perception of Hong Kong adolescents. It also included a part of measuring the participants’ demographic characteristics including age, gender, father educational level, mother educational level, family intactness, and family socioeconomic status (i.e., receiving Comprehensive Social Security Assistance (CSSA) or not). Families receiving CSSA in Hong Kong are commonly regarded as poor families.

#### 2.2.1. Perceived Character Traits (Part 1)

Materialism: A shortened version of the Chinese Adolescent Materialism Scale (CAMS) [50] was adopted in our study to measure participants’ perceived materialism of themselves. The original CAMS contained 21 items measuring adolescents’ materialism in different dimensions showing good reliability and validity [50]. In the present study, we used five items of the CAMS. A sample item was “I think that making money is more important than any other things”. All the items were answered in terms of a five-point Likert scale ranging from 1 (“Strongly disagree”) to 5 (“Strongly agree”). The Cronbach’s alpha value for perceived materialism of self was 0.81. Confirmatory factor analysis (CFA) demonstrated that the five items of the CAMS had acceptable goodness-of-fit (Comparative fit index (CFI) = 0.97, Tucker-Lewis index (TLI) = 0.94, root mean square error of approximation (RMSEA) = 0.08, standardized root mean residual (SRMR) = 0.03).

Egocentrism: We employed five items of the Chinese Adolescent Egocentrism Scale (CAES) to measure egocentrism of the participants [51]. The original CAES contained 14 items that possessed good psychometric properties [51]. One sample item was “My own benefits are more important than the benefits of other people”. These items were answered using a five-point Likert scale ranging from 1 (“Strongly disagree”) to 5 (“Strongly agree”). The Cronbach’s alpha value of the five-item CAES in our study was 0.77. Through CFA, these five items of the CAES showed acceptable goodness-of-fit (CFI = 0.98, TLI = 0.95, RMSEA = 0.09, SRMR = 0.02).

Moral Character: The participants’ perceived moral character was measured by a 15-item scale with excellent psychometric properties [10]. For each item, the participants were requested to indicate to what extent they possess the specific character trait on a five-point Likert scale ranging from 1 (“Strongly disagree”) to 5 (“Strongly agree”). The Cronbach’s alpha value in our study for moral character was 0.93. Acceptable goodness-of-fit of this scale was shown via CFA (CFI = 0.91, TLI = 0.89, RMSEA = 0.08, SRMR = 0.04).

#### 2.2.2. Perceived Well-Being (Part 2)

Empathy: The 11-item Empathy subscale of the Chinese version of the Interpersonal Reactivity Index (C-IRI) [52] was adopted to measure the participants’ empathy as an index of interpersonal well-being. A sample item in the Empathy subscale was “When I see someone being taken advantage of, I feel kind of protective towards them”. All items were measured in terms of a five-point Likert scale ranging from 1 (“Strongly disagree”) to 5 (“Strongly agree”). The Cronbach’s alpha value of the scale in this study was 0.78. CFA showed that the 11-item of the Empathy demonstrated fair goodness-of-fit (CFI = 0.96, TLI = 0.92, RMSEA = 0.09, SRMR = 0.03).

Life Satisfaction: Participants’ life satisfaction was assessed by the five-item Satisfaction with Life Scale (SWLS) [53]. The SWLS measures people’s global satisfaction with their lives. One sample item was “In most ways my life is close to my ideal”. Each item was measured in terms of a six-point Likert scale ranging from 1 (“Strongly disagree”) to 6 (“Strongly agree”). The scale was shown to have good reliability and validity in different cultural contexts [54,55]. The Cronbach’s alpha value of the five-item SWLS was 0.8. CFA showed that this scale showed good goodness-of-fit (CFI = 0.99, TLI = 0.98, RMSEA = 0.08, SRMR = 0.01).

#### 2.2.3. Perceived Social Behavior (Part 3)

Social Trust: The Research Team developed a scale to measure perceived social trust. The scale includes 11 items measuring adolescents’ trust of 11 social organizations, such as police, court, Hong Kong government [56]. All items were measured in terms of a four-point Likert scale ranging from 1 (“Very high level of distrust”) to 4 (“Very high level of trust”). The Cronbach’s alpha value for the participants’ perceived social trust of themselves was 0.80, indicating good reliability. CFA revealed that the 11-item Social Trust Scale demonstrated good goodness-of-fit (CFI = 0.98, TLI = 0.97, RMSEA = 0.05, SRMR = 0.02).

Social Responsibility of Self: A 15-item scale was developed to measure the participants’ perceived responsibility of themselves, such as studying hard, respecting the elders, self-control, and so on [10]. All the items were answered in terms of a five-point Likert scale ranging from 1 (“Very bad”) to 5 (“Very good”). The Cronbach’s alpha value for the participants’ perceived responsibility of themselves was 0.89. The one-factor model based on the 15-item scale showed good factorial validity via CFA (CFI = 0.93, TLI = 0.91, RMSEA = 0.07, SRMR = 0.05).

Prosocial Behavior: A five-item scale was adopted to measure the participants’ prosocial behavior in the past year (i.e., in the past 12 months). One sample item was “In the past year, I have donated money to charities (including flag sale)”. All the items were rated on a seven-point Likert scale ranging from 0 (“Never”) to 6 (“More than 10 items”). Higher score indicates higher prosocial behavior in the past year. The Cronbach’s alpha value of the scale in our study was 0.74. CFA findings on the one-factor model of the scale showed acceptable goodness-of-fit (CFI = 0.98, TLI = 0.94, RMSEA = 0.08, SRMR = 0.02).

Prosocial Attitude: A five-item scale was developed based on the Chinese Positive Youth Development Scale [57] to measure the participants’ prosocial attitude. One sample item was “I care about the unfortunate people in society”. All the items were answered in terms of a six-point Likert scale ranging from 1 (“Strongly disagree”) to 6 (“Strongly agree”). The Cronbach’s alpha value for the scale was 0.76. CFA finding supported the one-factor model of the Prosocial Attitude Scale (CFI = 0.98, TLI = 0.95, RMSEA = 0.08, SRMR = 0.02).

#### 2.2.4. Views on Child Development (Part 4)

Three items were developed to assess the participants’ views on child development: (1) “Do you agree ‘Children have to win at the starting line’?”; (2) “Do you agree ‘Children should not lose at the starting line’?”; (3) “Do you agree ‘It is more important for adolescents to have good moral character than excellent academic results’?” All items were answered in terms of a four-point Likert scale ranging from 1 (“Strongly disagree”) to 4 (“Strongly agree”).

#### 2.2.5. Perceived School Environment (Part 5)

Perceptions of School Climate: We adopted eight items from the Delaware School Climate Scale (DSCS) [58,59] to measure students’ perceptions of school climate. Among the eight items, four measured teacher-student relations (TSR), and the other four measured student-student relations (SSR). One sample item in TSR was “Teachers care about their students” and one sample item in SSR was “Students get along with one another”. All the items were measured in terms of a four-point Likert scale ranging from 1 (“Strongly disagree”) to 4 (“Strongly agree”). The Cronbach’s alpha values for TSR and SSR were 0.88 and 0.91, respectively. CFA showed that these two measures had good factorial validity based on the one-factor model (CFI = 0.99, TLI = 0.98, RMSEA = 0.08, SRMR = 0.01 for TSR; CFI = 0.99, TLI = 0.97, RMSEA = 0.09, SRMR = 0.01 for SSR).

Perceptions of Teachers’ Teaching Techniques: We selected 14 items from the Delaware Techniques Scale [58,60] to measure the participants’ perceived three types of teaching techniques of teachers in their schools. The three types of teaching techniques include Positive Behavior Techniques (PBT, 4 items), Punitive Techniques (PT, 5 items), and Social Emotional Learning Techniques (SELT, 5 items). Three sample items respectively for the three types of teaching techniques were “Students are often praised” (PBT), “Students are often punished” (PT), and “Teachers teach students to be responsible for their behaviors” (SELT). All the items were measured in terms of a four-point Likert scale ranging from 1 (“Strongly disagree”) to 4 (“Strongly agree”). The Cronbach’s alpha values for different teaching techniques were 0.75, 0.76, and 0.80, respectively. CFA findings supported the one-factor models associated with these measures (CFI = 0.98, TLI = 0.95, RMSEA = 0.08, SRMR = 0.02 for PBT; CFI = 0.99, TLI = 0.98, RMSEA = 0.05, SRMR = 0.01 for PT; CFI = 0.99, TLI = 0.98, RMSEA = 0.06, SRMR = 0.02 for SELT).

Perceptions of Moral Education in School: Four items were developed to assess the participants’ perceptions of moral education in current schools in Hong Kong. The first item assesses the participants’ perceived adequacy of moral education at their current school. The second and third items assess the participants’ perceived adequacy of knowledge about life skills they and their peers learned from the curriculum of their current schools. These three items were measured in terms of a four-point Likert scale ranging from 1 (“Very inadequate”) to 4 (“Very adequate”). The fourth item assesses the participants’ perceived necessity of acquiring life skills for Hong Kong adolescents, which was measured in terms of a four-point Likert scale ranging from 1 (“Very unnecessary”) to 4 (“Very necessary”).

#### 2.2.6. Perceptions of Hong Kong Adolescents (Part 6)

Materialism of Hong Kong Adolescents: The same five items used under Part 1 were used to assess one’s perception of materialism of Hong Kong adolescents in general. A sample item was “Hong Kong adolescents think that making money is more important than any other things”. All the items were answered in terms of a five-point Likert scale ranging from 1 (“Strongly disagree”) to 5 (“Strongly agree”). The Cronbach’s alpha value for perceived materialism of Hong Kong adolescents was 0.88. Acceptable factorial validity based on the one-factor model was found via CFA (CFI = 0.96, TLI = 0.93, RMSEA = 0.08, SRMR = 0.03).

Egocentrism of Hong Kong Adolescents: We used the same five items in Part 1 to understand one’s perception of egocentrism in Hong Kong adolescents. One sample item was “The benefits of Hong Kong adolescents in general are more important than the benefits of other people”. All five items were answered in terms of a five-point Likert scale ranging from 1 (“Strongly disagree”) to 5 (“Strongly agree”). The Cronbach’s alpha value for perceived Egocentrism of Hong Kong adolescents was 0.89. CFA showed that this scale had acceptable factorial validity based on the one-factor model (CFI = 0.96, TLI = 0.93, RMSEA = 0.08, SRMR = 0.03).

Moral Character of Hong Kong Adolescents: We employed the same 25 items used in Part 1 to assess 25 moral character attributes in adolescents in Hong Kong. For each item, the participants were requested to indicate to what extent they possess the specific character trait on a five-point Likert scale ranging from 1 (“Strongly disagree”) to 5 (“Strongly agree”). The Cronbach’s alpha value for measuring participants’ perception of HK adolescents was 0.95. The one-factor model based on the 25 items was supported via CFA (CFI = 0.95, TLI = 0.90, RMSEA = 0.08, SRMR = 0.04).

Moral Competence of Hong Kong Adolescents: Two items were developed to evaluate the participants’ perceived level of moral competence of Hong Kong adolescents. One item was “Do you think the level of moral competence of Hong Kong adolescents is high or low?”, which was answered in terms of a 5-point Likert scale ranging from 1 (“Very low”) to 5 (“Very high”). Another item was “Do you think the level of moral competence of Hong Kong adolescents is gradually going downwards, upwards, or similar to the past?”, which was rated on a 3-point Likert scale ranging from 1 (“Going downwards gradually”) to 3 (“Similar to the past”).

Perceived Psychosocial Competence of Hong Kong Adolescents: An 8-item scale was developed to measure the psychosocial competence of Hong Kong adolescents perceived by the participants, including moral competence, emotional competence, resilience, problem solving, having life goals, gratefulness, integrity and social competence. All items were measured in terms of a five-point Likert scale ranging from 1 (“Very weak”) to 5 (“Very strong”). The Cronbach’s alpha value for the scale was 0.88. CFA showed that the 11 items of the Perceived Psychosocial Competence of Hong Kong CFA findings supported the one-factor model of this scale (CFI = 0.96, TLI = 0.93, RMSEA = 0.08, SRMR = 0.03).

Social Trust of Hong Kong Adolescents: The same 11 items used in Part 3 were used to assess adolescents’ trust of 11 social organizations. All items were measured in terms of a four-point Likert scale ranging from 1 (“Very high level of distrust”) to 4 (“Very high level of trust”). The Cronbach’s alpha value for the participants’ perceived social trust of Hong Kong adolescents was 0.84. CFA gave support for the one-factor model of the 5-item scale (CFI = 0.96, TLI = 0.93, RMSEA = 0.08, SRMR = 0.03).

Social Responsibility of Hong Kong Adolescents: The same 15 items used in Part C were employed to assess participants’ perceived social responsibility of Hong Kong adolescents. All items were answered in terms of a five-point Likert scale ranging from 1 (“Very bad”) to 5 (“Very good”). The Cronbach’s alpha value for the participants’ perceived responsibility of Hong Kong adolescents was 0.93. The one-factor model based on the 15 items was supported by CFA (CFI = 0.96, TLI = 0.95, RMSEA = 0.08, SRMR = 0.03).

### 2.3. Data Analyses

As there are different groups of dependent variables, we conducted a MANOVA for a group of dependent variables first, followed by univariate ANOVAs. In all analyses, covariates, including student age, gender, parent educational level, family intactness, and family economic status, were first controlled [61].

Results of MANOVA showed that there was a significant overall difference between immigrant and non-immigrant student groups on the best linear combination of all dependent variables (Omnibus F = 2.068, *p* < 0.001, partial η^2^ = 0.069). As the omnibus F was significant, univariate ANOVAs were conducted for all measures. To avoid inflated Type 1 error, we adopted Bonferroni-corrected alpha level for each category of the dependent variables (i.e., dividing 0.05 by the number of tests in a category).

## 3. Results

Table 1 and Table 2 show the demographic characteristics of the sample and the descriptive statistics of the measures. Speaking overall, Chinese adolescent immigrants had more favorable perceptions of their own moral character, empathy, social trust, and school environment as well as characteristics of Hong Kong adolescents (moral character, social trust and social responsibility) than did Hong Kong adolescent non-immigrants (Table 3).

### 3.1. Perceived Character Traits (Part 1)

While the two groups of students scored similarly on perceived materialism and egocentrism, immigrant students scored significantly higher than non-immigrant students on perceived moral character based on Bonferroni-corrected alpha level (*p* = 0.017).

### 3.2. Perceived Well-Being (Part 2)

Using a Bonferroni-corrected alpha level (*p* = 0.025), the immigrant group scored significantly higher than the non-immigrant group on empathy.

### 3.3. Perceived Social Behavior (Part 3)

Based on a Bonferroni-corrected alpha level (*p* = 0.0125), immigrant students scored significantly higher than non-immigrant students on perceptions of social trust.

### 3.4. Views on Child Development (Part 4)

With regard to views on child development, ANCOVA results showed that the two groups did not differ in their views on child development based on the three items.

### 3.5. Perceived School Environment (Part 5)

With regard to school climate, ANCOVA results showed that the immigrant group scored significantly higher than the non-immigrant group on teacher-student relations (Bonferroni-corrected alpha level = 0.025). Regarding teacher techniques, the immigrant group perceived a higher usage of social emotional learning techniques and positive behavioral techniques as compared to non-immigrant students (Bonferroni-corrected alpha level = 0.017). In addition, immigrant students scored higher than non-immigrant students on perceptions of necessity of acquiring the life skills for adolescents.

### 3.6. Perceived Characteristics of Hong Kong Adolescents (Part 6)

Employing a Bonferroni-corrected alpha level (*p* = 0.00625), immigrant students scored significantly higher than non-immigrant students on their perceptions of moral character, social trust and social responsibility of adolescents in Hong Kong.

## 4. Discussion

The present study showed that mainland Chinese immigrant adolescents did not perform worse on most psychosocial indicators, and they in fact performed better on moral character, empathy and social trust. They also had more favorable perceptions of the school environment and characteristics of adolescents in Hong Kong (including moral character, social trust and social responsibility). Taken as a whole, while the findings provide support for the healthy immigration hypothesis, there is no evidence for the migration morbidity hypothesis.

In contrast to previous studies, the present study comprehensively compared mainland Chinese immigrant adolescents and native Hong Kong adolescents over six domains. First, our results showed that mainland Chinese immigrant adolescents scored significantly higher than non-immigrant students on perceived moral character, while the two groups of students scored similarly on perceived materialism and egocentrism. One possible explanation is that those from mainland China may give heavier weight on moral character because mainland China strongly emphasizes traditional Chinese virtues and Socialist ideologies. In fact, moral education forms an important part of the formal curriculum in mainland China. These differences may explain why immigrant adolescents scored higher than adolescent non-immigrants over moral character attributes.

Second, we compared the differences of mainland Chinese immigrant adolescents and non-immigrants on perceived well-being. The results showed that there was no difference in life satisfaction between mainland Chinese immigrant students and the non-immigrant students, but Chinese adolescent immigrants scored significantly higher than the non-immigrant group on empathy. These results imply that the psychosocial development of mainland Chinese immigrant adolescents is not hindered by the environmental changes, but on the contrary, they were able to adapt to the environmental changes well. This difference suggests that immigrants in China would be more able to take the perspective of others [62].

Third, we compared the differences of mainland Chinese immigrant adolescents and non-immigrants on their perceptions of social behaviors. Previous research has questioned that the negative social behavioral effects of migration are not really a result of migration, but rather the acculturation pressures of migration [3,4,63]. The present study further proves that at least the prosocial behavior of immigrants will not be adversely affected by migration. In addition, the findings suggested that the mainland Chinese immigrant students scored significantly higher than non-immigrant students on social trust, which is an important foundation to develop positive social attitudes [64,65].

Fourth, we compared the views of mainland Chinese immigrant adolescents and non-immigrants on their views on child development. The results showed that the two groups did not differ in their views of “children have to win at the starting line” and “children should not lose at the starting line”. This result is contrary to the common myth that immigrants are more instrumental in their aspirations. The present finding suggests that migration does not increase the opportunistic mindset of mainland immigrant adolescents. Instead, they are able to view the impact of the starting line on personal development in the same way as non-immigrants. The results also showed that there was no significant difference between the two groups on the item “It is more important for adolescents to have good moral character than excellent academic results” (i.e., instrumental attitude).

Fifth, we compared the differences of mainland Chinese immigrant adolescents and non-immigrants on perceived school environment. Our study found that mainland Chinese students were well-integrated into schooling in Hong Kong in terms of teacher-student relationships and perceptions of teachers’ use of social-emotional learning techniques and positive behavior techniques compared to non-immigrant students. These findings are in contrast to the findings that immigrant students showed school adjustment problems [13,42,43]. There are two possible explanations of the findings. First, teachers may pay special attention to immigrant students, hence contributing to the positive findings. Second, as the school system in Hong Kong adopts a more student-centered approach (i.e., individualistic focus) as compared to that in mainland China, immigrant students would have better perceptions of their teachers.

Finally, we compared the difference of mainland Chinese immigrant adolescents and non-immigrants on the perceptions of Hong Kong adolescents in general. Results showed that mainland Chinese immigrant adolescents have significantly higher scores on their perceptions of moral character, responsibility and social trust of Hong Kong adolescents than did non-immigrant adolescents, while the two groups showed no significant differences in other aspects. For immigrant adolescents, non-immigrant adolescents as a reference group have a very important role in guiding their psychosocial development in receiving community. Generally speaking, the more positively immigrant adolescents perceive the psychosocial development of non-immigrant adolescents, the more they can acquire the mainstream values. Our study found that mainland Chinese immigrant adolescents perceive the psychosocial development of Hong Kong adolescents more positively than did non-immigrants, which has a very important practical value for fostering integration of immigrant adolescents in Hong Kong.

There are three theoretical contributions of the present study. First, the findings provide support for the healthy migration hypothesis within the context of internal immigration. The present findings expand the theoretical scope of the healthy migration hypothesis particularly within the context of internal migration, particularly in an Asian context. Second, the study suggests that it is desirable to consider the validity of the migration morbidity hypothesis and healthy migration hypothesis with reference to a wide range of indicators. In fact, besides negative mental health measures such as internalizing and externalizing problems, inclusion of other measures including social behavior and social perception would help to enrich these two hypotheses. Third, the findings of the present study have some value for research on the psychosocial development of international migration. Although previous studies have focused on the psychosocial adaptation of transnational and cross-cultural immigrants, most of them have not considered the effects of different cultures and ethnic identities on immigrants’ psychosocial adaptation. This study was conducted in the same country and cultural context with the finding that the two groups shared similar profiles in most indicators. This highlights the important influence of culture and ethnic identity on immigrants’ psychosocial adaptation. In addition, immigrants’ acculturation strategies, whether immigrants positively integrate into the host society or maintain their unique cultural and ethnic identities, are also likely to positively influence their psychological and behavioral adjustment outcomes. Thus, it is necessary to include factors such as cultural similarity, ethnic identity and acculturation strategies in the analysis of future studies.

Practically, the findings demystify the common myth that adolescent immigrants would necessarily have adjustment problems. The findings are important for social integration of adolescent immigrants as educators, and social workers can use them for public education purposes. On the one hand, we should believe that adolescent immigrants have the ability to cope with the various stresses of living in the host country, and in practice, we should focus more on how to stimulate this ability of immigrants, which can promote their cultural integration. One common method is empowerment, which may be defined as a process to increase personal, interpersonal and political power so that they can take actions to improve their life situation [66]. On the other hand, the psychosocial and behavioral aspects of the host adolescent have an important guiding role, and in practice, we should encourage the interaction of immigrant adolescents with native adolescents so that they can acquire mainstream values and behavioral patterns. For public education purposes, persistent negative stereotypes are an important barrier to intergroup integration [67,68,69], and the findings of the present study help encourage host country residents to abandon the perceptions of immigrant disadvantage in order to reduce negative stereotypes of host country residents’ psychosocial and behavioral perceptions of immigrants, and enhance social integration between the two groups. Finally, as different stakeholders viewed that Chinese high school students lacked life skills such as empathy and social competence [30,70], there is a need to promote social competence (e.g., respect for diversity, social competence and empathy) in high school students via evidence-based youth enhancement programs such as the Project P.A.T.H.S. [71], which can help to integrate immigrant and local adolescents in Hong Kong.

There are three limitations of this study. First, self-report measures were used in this study. While this approach is commonly used in the immigration literature [2,6,18,28], it would be helpful if the developmental outcomes could be established by the significant-others of the students. Second, a cross-sectional design was used, which cannot fully capture the cause–effect relationship between migration and developmental outcomes. In future, a longitudinal design comparing immigrants and non-immigrant adolescents over time would be helpful because previous studies have found that whether immigrant adolescents experience worse psychological and behavioral outcomes than the natives is related to the length of time he or she has lived in the host country [3]. Although our project asked the Chinese immigrant adolescents about the length of residence in Kong Hong, the information given by the participants is not exact (e.g., whether they were first-generation migrants). As previous studies have suggested that increased externalizing symptoms may be interpreted as signs of migrant children’s attempts to adjust to societal acculturation and adaptation to the environment [3,72], it would be interesting to examine whether length of immigration and “generation” of migrants would be related to different developmental outcomes. Finally, there is a need to comprehensively assess the psychological well-being in immigrant Chinese adolescents in Hong Kong. Besides life satisfaction and empathy, other measures of well-being, such as depression and hopelessness, would help to illuminate the relationship between immigration and adolescent well-being [8,9].

## 5. Conclusions

In conclusion, in contrast to previous studies that have focused more on international migration, this study focused on the impact of internal migration within the same country on the psychological and social behavior of adolescents. Unlike previous studies that focused on psychological or social behavior, this study provides a more comprehensive assessment of the psychological and social behavioral outcomes related to migration. The findings revealed that Chinese immigrant adolescents and non-immigrant adolescents shared similar profiles in most indicators, and the perceived moral character, empathy, social trust and social responsibility of the Chinese immigrant adolescents were significantly higher than those of native adolescents. This means that if we compare the psychological and social behaviors of immigrants and native adolescents from a single dimension, we cannot fully and truly assess the impact of migration on immigrants, and in some cases more negative or more positive results may just be a coincidence.

## Figures and Tables

**Table 1:** Demographic variables of Chinese immigrant and non-immigrant student groups.

Demographic Variables	Chinese Immigrant Student Group (*n* = 590)	Non-Immigrant Student Group (*n* = 1798)	*t* Value	*p* Value
	Mean (SD)	Mean (SD)		
Age	15.14 (1.87)	14.23 (1.68)	11.626	0.000
	*n* (%)	*n* (%)		
Gender				
Female (%)	330 (57.7%)	896 (51.2%)	2.703	0.007
Male (%)	242 (42.3%)	854 (48.8%)		
Father Educational Level				
No Formal Education and Llliterate	3 (0.7%)	6 (0.5%)	0.601	0.547
No formal Education but Literature	9 (2.0%)	18 (1.4%)	1.045	0.296
Primary 1–5	40 (8.9%)	70 (5.3%)	2.906	0.003
Primary 6	61 (13.5%)	135 (10.2%)	2.175	0.029
Secondary 1–3	115 (25.5%)	290 (21.9%)	1.889	0.059
Secondary 4–5	61 (13.5%)	235 (17.8%)	−1.747	0.081
Secondary 6–7	94 (20.8%)	284 (21.5%)	0.079	0.937
Sub-degree Level	20 (4.4%)	65 (4.9%)	−0.256	0.797
Bachelor’s Degree or Higher	48 (10.6%)	219 (16.6%)	−2.708	0.007
Mother Educational Level				
No Formal Education and Illiterate	6 (1.3%)	12 (0.9%)	0.851	0.394
No Formal Education but Literature	10 (2.2%)	24 (1.8%)	0.640	0.522
Primary 1–5	48 (10.6%)	67 (5.0%)	4.356	0.000
Primary 6	85 (18.8%)	127 (9.6%)	5.473	0.000
Secondary 1–3	140 (31.0%)	249 (18.7%)	5.674	0.000
Secondary 4–5	46 (10.2%)	283 (21.3%)	−4.879	0.000
Secondary 6–7	68 (15.0%)	322 (24.2%)	−3.648	0.000
Sub-degree Level	30 (6.6%)	82 (6.2%)	0.522	0.601
Bachelor’s Degree or Higher	19 (4.2%)	163 (12.3%)	−4.662	0.000
Family Intactness				
Intact	353 (61.1%)	1403 (79.0%)	−8.707	0.000
Non-intact	225 (38.9%)	374 (21.0%)		
Family Socio-economic Status				
Receiving CSSA	68 (14.8%)	166 (11.2%)	2.052	0.040
Not Receiving CSSA	392 (85.2%)	1314 (88.8%)		

**Table 2:** Descriptive statistics for the main dependent variables.

Domains and Related Measures	Mean	SD	Min	Max
**Perceived Character Traits**				
Student-Materialism	2.36	0.79	1	5
Student-Egocentrism	2.59	0.71	1	5
Student-Moral Character	3.70	0.53	1	5
**Perceived Well-Being**				
Students’ Empathy	3.52	0.51	1	5
Students’ Life Satisfaction	3.35	1.14	1	6
**Perceived Social Behavior**				
Student-Social Trust	2.54	0.44	1	4
Student-Responsibility	3.43	0.57	1	5
Student’s Prosocial Behavior in Past 12 Months	1.44	1.07	0	6
Students’ Prosocial Attitudes	4.33	0.80	1	6
**Views of Child Development**				
Agreement on “Children Have to Win at The Starting Line.”	2.02	0.86	1	4
Agreement on “Children Should Not Lose at The Starting Line.”	2.29	0.87	1	4
Agreement on “It is more important for adolescents to have good moral character than excellent academic results.” (single item)	3.26	0.74	1	4
**Perceived School Environment**				
*School Climate*				
Student-Student Relations	2.93	0.58	1	4
Teacher-Student Relations	2.90	0.56	1	4
*Teacher Technique Scale*				
Teacher Use of Punitive Pechniques	2.29	0.59	1	4
Teacher Use of Positive Behavioral Techniques	2.74	0.52	1	4
Teacher Use of Social Emotional Learning Techniques	2.91	0.48	1	4
*Perceptions of Moral Education in School*				
Adequacy of Moral Education in School	2.74	0.70	1	4
Adequacy of Your Knowledge About Life Skills Learnt From The Current Curriculum in School	2.70	0.75	1	4
Adequacy of Adolescents’ Knowledge About Life Skills Learnt From The Current Curriculum in School	2.52	0.76	1	4
Necessity of Acquiring Life Skills for Adolescents	3.22	0.66	1	4
**Perceptions of Hong Kong Adolescents**				
Adolescents-Materialism	3.16	0.89	1	5
Adolescents-Egocentrism	3.46	0.80	1	5
Adolescents-Moral Character	3.07	0.60	1	5
Perception of Level of HK Adolescents’ Moral Competence (Single Item)	2.98	0.76	1	5
Perceptions of Trend of HK Adolescents’ Moral Competence (Single Item)	2.07	0.92	1	3
Adolescents-Psychosocial Competence	2.94	0.66	1	5
Adolescents-Social Trust	2.47	0.50	1	4
Adolescents-Responsibility	3.02	0.63	1	5

**Table 3:** ANCOVA results—Differences between Chinese Immigrant and Non-Immigrant Students with Student Age, Gender, Father and Mother Educational Level, Family Intactness, and Economic Status as Covariates.

Variables	Non-Immigrant M (SD)	Chinese Immigrant M (SD)	*F*	*p*	Partial Eta Squared
Omnibus Analysis			2.068	0.001	0.069
**Perceived Character Traits**					
Student-Materialism	2.32 (0.79)	2.47 (0.76)	0.389	0.533	0.000
Student-Egocentrism	2.57 (0.70)	2.59 (0.69)	0.035	0.852	0.000
Student-Moral Character	3.71 (0.52)	3.79 (0.54)	8.781	0.003	0.007
**Perceived Well-Being**					
Students’ empathy	3.55 (0.52)	3.62 (0.46)	5.089	0.024	0.004
Students’ life satisfaction	3.38 (1.15)	3.20 (1.07)	0.033	0.856	0.000
**Perceived Social Behavior**					
Student-Social Trust	2.51 (0.42)	2.58 (0.43)	16.654	0.000	0.013
Student-Responsibility	3.45 (0.55)	3.48 (0.56)	3.494	0.062	0.003
Student’s prosocial behavior in past 12 months	1.54 (1.12)	1.35 (0.88)	4.528	0.034	0.003
Students’ prosocial attitudes	4.40 (0.78)	4.49 (0.73)	5.868	0.016	0.005
**Views of Child Development**					
Agreement on “Children have to win at the starting line.”	1.94 (0.86)	2.05 (0.89)	0.087	0.769	0.000
Agreement on “Children should not lose at the starting line.”	2.22 (0.89)	2.31 (0.87)	0.234	0.629	0.000
Agreement on “It is more important for adolescents to have good moral character than excellent academic results.” (single item)	3.30 (0.73)	3.27 (0.77)	1.389	0.239	0.001
**Perceived School Environment**					
*School Climate*					
Student-student relations	2.93 (0.57)	2.95 (0.56)	1.540	0.215	0.001
Teacher-student relations	2.85 (0.57)	2.98 (0.51)	14.748	0.000	0.011
*Teacher Technique Scale*					
Teacher use of punitive techniques	2.27 (0.61)	2.26 (0.57)	0.002	0.969	0.000
Teacher use of positive behavioral techniques	2.71 (0.52)	2.78 (0.49)	6.787	0.009	0.005
Teacher use of SEL techniques	2.89 (0.49)	2.95 (0.46)	9.853	0.002	0.008
*Perceptions of Moral Education in School*					
Adequacy of moral education in school	2.69 (0.71)	2.66 (0.67)	0.766	0.382	0.001
Adequacy of your knowledge about life skills learnt from the current curriculum in school	2.67 (0.77)	2.59 (0.76)	0.230	0.880	0.000
Adequacy of adolescents’ knowledge about life skills learnt from the current curriculum in school	2.49 (0.78)	2.47 (0.70)	2.128	0.145	0.002
Necessity of acquiring life skills for adolescents	3.21 (0.69)	3.33 (0.61)	5.163	0.023	0.004
**Perceptions of Hong Kong Adolescents**					
Adolescents-Materialism	3.20 (0.87)	3.18 (0.91)	1.347	0.246	0.001
Adolescents-Egocentrism	3.50 (0.79)	3.49 (0.84)	0.979	0.323	0.001
Adolescents-Moral Character	3.04 (0.60)	3.18 (0.62)	9.352	0.002	0.007
Perception of level of HK adolescents’ moral competence (single item)	2.99 (0.78)	3.01 (0.79)	0.131	0.718	0.000
Perceptions of trend of HK adolescents’ moral competence (single item)	2.00 (0.93)	1.97 (0.91)	0.002	0.963	0.000
Adolescents-Psychosocial Competence	2.90 (0.65)	2.99 (0.63)	6.483	0.011	0.005
Adolescents-Social Trust	2.39 (0.48)	2.54 (0.52)	28.260	0.000	0.021
Adolescents-Responsibility	3.00 (0.63)	3.12 (0.66)	8.055	0.005	0.006

Note. For each group of measures, Bonferroni correction was carried out (i.e., 0.05/number of tests).

## Data Availability

The data presented in this study are available on request from the corresponding author. The data are not publicly available due to privacy.
